# Diagnostic complexity of rifampicin-induced coagulopathy in a patient with spontaneous muscle bleeding: A case report

**DOI:** 10.1097/MD.0000000000026234

**Published:** 2021-07-02

**Authors:** Domagoj Vučić, Katica Cvitkušić-Lukenda, Ivica Dunđer, Krešimir Gabaldo, Marijana Knežević-Praveček, Blaženka Miškić

**Affiliations:** a Department for Internal Medicine, General Hospital ”dr. Josip Benčević,” Andrije Štampara 42, Slavonski Brod, Croatia; b Postgraduate Interdisciplinary Study of Molecular Bioscience, University of Josip Juraj Strossmayer, Cara Hardijana 8/A, Osijek, Croatia; c Faculty of Dental Medicine and Health, University of Josip Juraj Strossmayer, Crkvena 21, Osijek, Croatia; d Faculty of Medicine, University of Josip Juraj Strossmayer, Josipa Huttlera 4, Osijek, Croatia.

**Keywords:** case report, coagulopathy, disseminated intravascular coagulation, infective endocarditis, rifampicin

## Abstract

**Introduction:**

Rifampicin is currently used to treat various bacterial infections, with the most significant application in the treatment of tuberculosis. Dose-independent side effects of the drug can lead to the development of various coagulation disorders, among which disseminated intravascular coagulation is the most dangerous. The mechanism of coagulopathy itself is multifactorial, but it is thought to be mediated by an immune response (formation of antigen-antibody complexes) and consequent damage to platelets and the vascular endothelium.

**Patient concerns:**

A 66-year-old woman, with numerous comorbidities including chronic renal failure, condition after implantation of a permanent pacemaker, and a positive blood culture for *Staphylococcus aureus*, presented with spontaneous bleeding in the muscle wall, and in the clinical picture of hemorrhagic shock.

**Diagnosis:**

Knowing the multifactorial mechanism of rifampicin-induced coagulopathy, possible factors were considered, such as infections, comorbidities, drug use and drug-drug interactions, pathological laboratory parameters, and coagulograms. Clinical presentation of abdominal pain and intra-abdominal mass, with laboratory verification of prolonged activated partial thromboplastin time and computed tomography-proven hematoma suspected of acute bleeding, redirects clinical suspicion of drug-induced coagulopathy.

**Interventions:**

By discontinuing rifapicin and administering vitamin K and fresh frozen plasma, normalization of laboratory coagulation parameters was achieved. Bleeding from the muscle wall required correction of acute anemia with red cell concentrates, surgical intervention, and additional antibiotic therapy for secondary infection of the operative wound.

**Outcomes:**

At the end of 6 weeks of antibiotic (antistaphylococcal) therapy (due to the basic suspicion of possible infectious endocarditis), the normalization of inflammatory parameters occurred with a sterile control blood culture and a normal coagulogram.

**Conclusion:**

Clinicians should be aware of the possible side effects of the administered drugs, especially taking into account the overall clinical picture of a patient, including comorbidities and possible drug interactions.

## 1 Introduction

Rifampicin, an antibiotic agent that is currently used to treat a variety of bacterial infections, including infections mediated by *Mycobacterium* spp., gram-positive cocci, and certain gram-negative pathogens. Its application led to a reduction in the total duration of antibiotic treatment of tuberculosis to 6 months in combination with pyrazinamide.^[1]^ In addition, it has been applied in the treatment of meningitis and brain abscesses (due to its ability to cross the blood-brain barrier), endocarditis, osteomyelitis, prosthetic joint infections (most often in hip and knee replacement), and implanted cardiac devices such as permanent pacemakers and cardioverter-defibrillator devices.^[2–4]^ Of the nonbacterial indications, it is used in the treatment of pruritus as a second-line therapy (along with cholestyramine).^[5]^ Owing to its susceptibility to bacterial mutations and the potential development of resistance, it is now used in combination with other antibacterial agents (in exceptional situations as a monotherapy for prophylactic purposes when bioburden is expected to be low). ^[3]^

**Figure 1:**
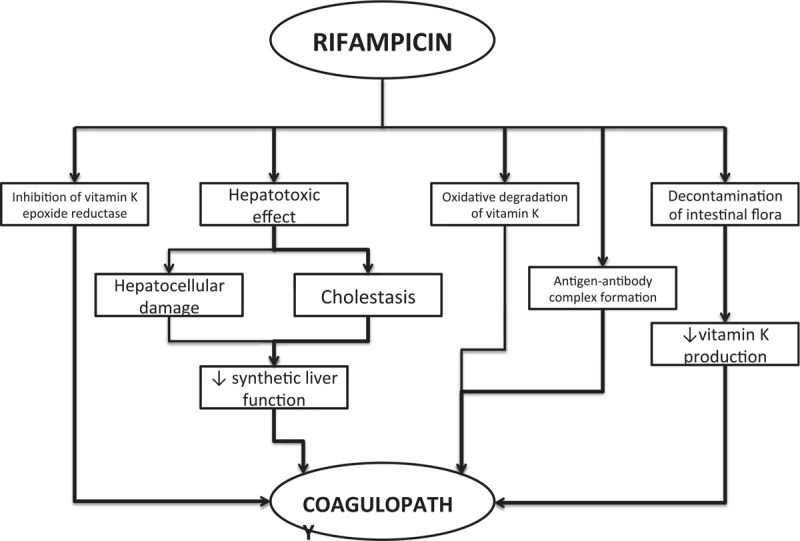
Pathophysiological complexity of rifampicin-induced coagulopathy. ↓ – decreasing.

The drug's side effects can be classified as dose-dependent and dose-independent.^[6]^ Dose-dependent side effects include hepatotoxicity (mediated by the development of toxic hepatitis), nausea, loss of appetite, and yellow discoloration of body fluids (urine, tears, saliva, etc). The development of urticaria (or any other form of a hypersensitivity reaction), acute renal failure, thrombocytopenia, and hemolysis are dose-independent side effects. The pathophysiological mechanism of coagulopathy, from less dangerous conditions such as milder thrombocytopenia to life-threatening disorders such as disseminated intravascular coagulation (DIC), is complex and multifactorial. It is known that rifampicin-induced coagulopathy occurs because of the lack of coagulation factors dependent on vitamin K (II, VII, IX, and X). Deficiency of the same coagulation factors occurs due to reduced synthetic liver function mediated by the hepatotoxic effect of the drug, reduced production of vitamin K by the intestinal flora (decontamination when administered orally), inhibition of vitamin K epoxide reductase (similar to warfarin application), and stimulation of vitamin K degradation.^[7–10]^ An additional factor in the development of coagulopathy is the formation of an antigen-antibody complex (mediated by immunoglobulins M and G) with the consequent development of systemic intravascular coagulation (consumption coagulopathy) (Fig. 1).^[11]^ Which of the described pathophysiological mechanisms will lead to the development of coagulopathy is difficult to predict, but some conditions such as pre-existing liver diseases (liposoluble drugs with bile excretion), drug-drug interactions (increased cytochrome P450 oxidase expression), chronic renal failure, and hypoalbuminemia (the ratio of unbound and bound forms of the drug) may increase the likelihood of adverse effects.

In the manuscript, the authors present a case report of a patient with the development of rifampicin-induced coagulopathy, presented in an unusual and unrecorded way, spontaneous rupture of the muscle wall. A review of available databases has so far reported a small (sporadic) number of cases of rifampicin-induced coagulopathy and never with spontaneous muscle bleeding.

## 2 Case report

**Figure 2:**
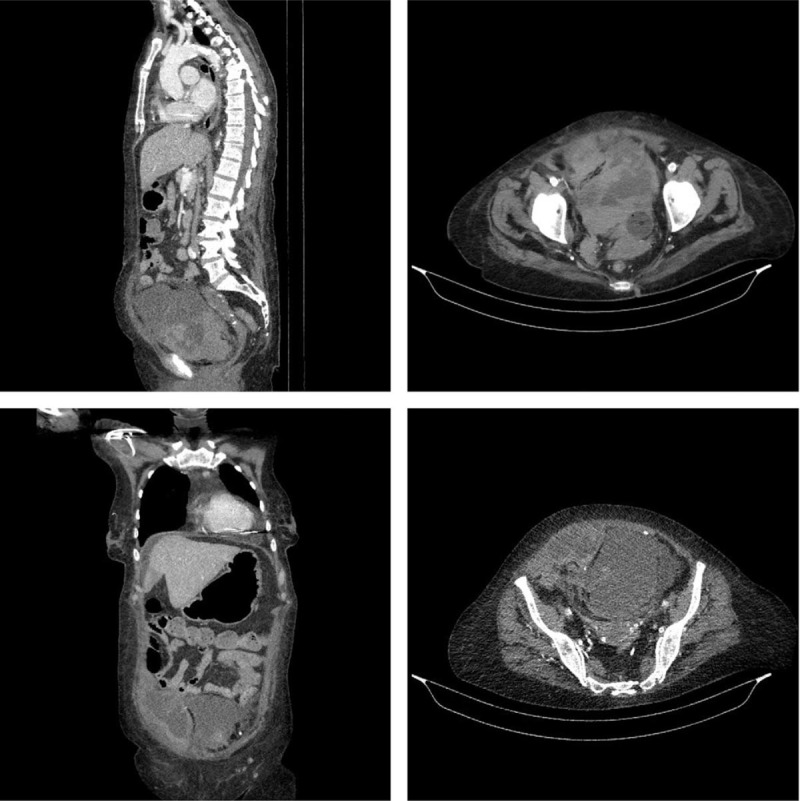
Multidetector computed tomography of abdomen and pelvis with intravenous contrast application, reveals hematoma in the right rectus abdominal muscle and large preperitoneal hematoma in Retzius space, displacing the bladder dorsally.

A 66-year-old caucasian female patient was admitted to the cardiology department of our institution on suspicion of an abscess in the right pectoral region, at the site of implantation of a permanent pacemaker and possible infective endocarditis (IE). Hospitalization was preceded by surgical treatment of the wound at another institution. Furthermore, antibiotic therapy with amoxicillin + clavulanic acid in a reduced dose (500 + 100 mg intravenously once daily or twice daily on hemodialysis days) was started in the same facility, according to the antibiogram of the wound culture, in which *Staphylococcus aureus* was isolated. The patient had previously been treated for chronic kidney disease (terminal uremia stage) and had been on a chronic hemodialysis program (3 times a week), type 2 diabetes (insulin-independent), arterial hypertension, anemia of chronic disease, hyperlipidemia, and the condition after implantation of a single-chamber electrostimulator in 2016, due to the development of an atrioventricular block (2nd degree; Mobitz II). The patient's family history was negative for cardiovascular diseases and diabetes mellitus. In regular therapy, the patient received sevelamer carbonate 800 mg orally every 12 hours, methoxy polyethylene glycol-epoetin beta of 200 mg (applied subcutaneously, once a month), nebivolol 5 mg, trandolapril 2 mg, and patoprazole 20 mg in the morning; lercanidipine 20 mg and atorvastatin 20 mg in the evening, and repaglinide 2 mg before main meals (3 times daily). Clinically, in the area of the right pectoral region (at the site of the previously implanted permanent pacemaker), redness of the skin was present, and subcutaneous fluctuation was painful to the touch. No other clinical features were observed. Laboratory findings upon admission showed increased leukocytes of 14.65 × 10^9^/L (normal value 3.4–9.5) with neutrophilia 83.3% (normal value 44%–72%), C-reactive protein 246.9 mg/L (normal value up to 5), procalcitonin 58.1 ng/mL (normal value up to 0.5), creatinine 446 μmol/L (normal value up to 90), urea 13.3 mmol/L (normal value 2.8–8.3), lactate dehydrogenase 297 U/L (normal value <247), gamma-glutamyltransferase 170 U/L (normal value 9–35), alkaline phosphatase 551 U/L (normal value 20–153), and normal values of erythrocytes, hemoglobin, mean corpuscular volume, platelets, sodium, potassium, aspartate, and alanine aminotransferase. Urine was unremarkable. Acid-base analysis of venous blood showed no signs of metabolic acidosis: pH 7.481 (normal value 7.35–7.43), partial pressure of carbon dioxide 4.94 kPa, and bicarbonate 27.0 mmol/L (normal value 10–11.3). The patient was also examined by an infectologist who, due to the fundamental suspicion of IE, recommended the introduction of parenteral antibiotic therapy of flucloxacillin 2 g intravenously every 6 hours, metronidazole 500 mg intravenously every 12 hours, and rifampicin 300 mg orally every 12 hours. Before starting antibiotic therapy, blood was sampled for aerobic and anaerobic blood culture and urine for urine culture. Transthoracic echocardiography showed normal left ventricular contractility, ejection fraction of 65%, no cavity dilation, and normal valvular apparatus, with no criteria for pulmonary hypertension, and no visible vegetation suggestive of IE. Electrostimulator analysis showed that the patient was independent of pacemaker stimulation (sensing 95%, pacing 5%, and with no asystolic pauses recorded). On the second day of hospitalization, the site of the previously implanted pacemaker was approached, the wound was cleaned with hydrogen peroxide, the electrode was disconnected from the generator, closed with a plug, and then fixed, after which the generator was removed. Upon the arrival of positive blood culture for *S aureus* (penicillin-resistant), parenteral antibiotic therapy was continued with a laboratory-present decrease in inflammatory parameters. The patient tolerated the therapy well and did not report any side effects. Furthermore, on the 15th day of hospitalization, the patient complained of sudden pain in the lower right abdomen. On re-examination, a hard mass was palpated, painful to the touch, and without propagation to other body parts. The patient had low blood pressure and cold extremities, causing suspicion of hemorrhagic shock. In laboratory, a significant decrease in blood counts was recorded; hemoglobin 89 g/L (normal value 119–157), erythrocytes 2.99 × 10^12^/L (normal value 3.86–5.08), hematocrit 25.5% (normal value 35.6%–47.0%), and platelets 299 × 10^9^/L (normal value 158–424). Abdominal ultrasound showed an inhomogeneous zone of 13 to 14 cm in diameter in the lower right abdomen. Emergency computed tomography of the abdomen was performed, which confirmed heterogeneous formation in the anterior abdominal wall of the lower right abdomen and in the small pelvis, without post-contrast imbibition, corresponding to hematoma (Fig. 2). A coagulogram was made by which it was observed prolonged activated partial thromboplastin time 42.48 seconds (normal value 22.6–31), prothrombin time 38.97% (normal value 70%–120%), International Normalized Ratio of 1.95 (normal value 1.0–1.2), fibrinogen 5.75 g/L (normal value 1.5–4.5), thrombin time 24.38 seconds (normal value 15–21). Rifampicin-induced coagulopathy was suspected to be the cause of spontaneous bleeding, which is why the drug was excluded and the rest of the parenteral antibiotic therapy was continued. Therapeutically, 2 ampoules of vitamin K (phytomenadionum 10 mg/mL), 1 fresh frozen plasma, and 3 doses of red cell concentrates were administered intravenously, resulting in the normalization of coagulogram and complete blood count. A surgeon was consulted, who indicated a transfer to the Department of Vascular Surgery. Intraoperatively, a hematoma was observed in the area of the rectus abdominis muscle of the lower right abdomen and signs of spontaneous bleeding from the right inferior epigastric artery. The artery was ligated, and the wound toilet was made at the bleeding site without access to the intraperitoneal cavity. At the Department of Vascular Surgery on the 18th day after the operation, a resurgence of inflammatory parameters was noted, and parenteral treatment with imipenem and oral vancomycin was initiated because of the suspicion of a secondary bacterial infection of the wound. During the total stay of the patient in our institution, a total of 6 weeks of antistaphylococcal antibacterial treatment was carried out, and the patient was discharged home with a recommendation to take the current chronic therapy with regular check-ups by cardiologists and surgeons.

## 3 Discussion

DIC represents a hyperactive state of blood with increased consumption of platelets and clotting factors, which can ultimately result in embolic events or bleeding.^[12]^ The clinical picture depends on the course of coagulopathy, so chronic conditions such as malignancy (lasting weeks or months) are dominated by thrombus formation and embolic incidents. In contrast, in the acute development of DIC (eg, in sepsis), patients are most often presented with various clinical syndromes, from spontaneous bleeding to hemorrhagic shock. Therefore, when making a diagnosis and selecting the best therapeutic approach, it is necessary to clarify the underlying disease, given that DIC is likely to be a consequence of the same. In 2001, the Scientific and Standardization Committee of the International Society for Thrombosis and Hemostasis introduced a scoring system (criteria) for the preclinical diagnosis of DIC, distinguishing 2 subtypes of the disease: overt and non-overt disease subtypes.^[13]^ The early form, or so-called non-overt DIC, is a milder form of hemolytic dysfunction that, unlike overt DIC, does not reach the decompensated state. Two additional scoring systems are in use: the Japanese Ministry of Health, Labor and Welfare score, and the Japanese Association of Acute Medicine score.^[14,15]^ There is still no uniform view for the use of a particular scoring system, as each has its advantages and disadvantages. There are 4 basic types of DIC (bleeding, organ failure, massive bleeding, and non-symptomatic type) that can occur as a result of the course of the vector between hypercoagulation and hyperfibrinolysis.^[16]^ Bleeding type (predominance of hyperfibrinolytic vector) develops most often in conditions such as leukemia, aortic aneurysms, and obstetric diseases. In contrast, organ failure type (predominance of hypercoagulation vector) occurs in infectious diseases such as sepsis, while major bleeding type (dominance of both vectors) occurs in patients with major bleeding after major surgeries or in obstetric diseases. Finally, the non-symptomatic type (both vectors are weak) or simply pre-DIC is not manifested by clinical symptoms or signs but by abnormal laboratory findings.

DIC is thought to be induced by rifampicin as a result of immune activation, formation of antigen-antibody complexes (rifampicin-erythrocyte or platelet complexes), activation of complement, and consequent destruction of erythrocytes and platelets, damaging the vascular endothelium which ultimately results in intravascular coagulation.^[11]^ Furthermore, intermittent administration of rifampicin is considered to increase the risk of developing DIC due to repeated activation of the immune system, and patients with daily administration of the drug may have a greater tolerance for the development of an immune response.^[17,18]^

This is the first reported case of encountering rifampicin-induced coagulopathy in our institution, and to the best of our knowledge, this is the first reported case of spontaneous bleeding from a muscle wall. To resolve differential diagnostic doubts, we were guided by a multifactorial approach, as shown in Figure 1. Since the patient was not on anticoagulant therapy, with no reported traumatic injury or pre-existing liver disease (as a possible cause of hypoprothrobinemia or hypoalbuminemia), we suspected drug-induced coagulopathy. Successful treatment by drug exclusion and coagulogram correction (vitamin K and fresh-frozen plasma) suggests that coagulopathy is indeed caused by vitamin K deficiency. In addition, during the initial determination of the coagulogram, due to the technical shortcomings and lack of reagents for D-dimer, we were not able to determine the International Society for Thrombosis and Hemostasis criteria for DIC in the patient.^[13]^ In this case, the patient was infected, with positive blood culture for *S aureus*, with elevated procalcitonin, and without meeting the clinical criteria for sepsis or systemic inflammatory response syndrome.^[19,20]^ Given that DIC, which underlies the infection (or sepsis), is most often presented as an organ failure type, and not as a bleeding type, we cannot conclude with certainty that the DIC was underlying the development of bleeding, or that it may have been a non-symptomatic type of DIC.

It is known that tubulointerstitial kidney injury, mediated by immune hyperactivity (anti-rifampicin antibodies) with rifampicin can lead to acute renal failure, but the possible impact of end-stage renal disease and hemodialysis on the development of coagulopathy is unknown.^[21]^ An increased tendency to bleeding in patients with uremia occurs under the influence of several factors, including platelet dysfunction, impaired platelet-endothelial interaction, anemia, and abnormal nitric oxide production.^[22]^ In addition, the use of drugs with anticoagulant or antiplatelet properties and whose clearance primarily depends on renal function (predominantly hydrosoluble drugs) may lead to an increased tendency to bleeding in such patients. Approximately 80% of rifampicin in the blood is bound to proteins (predominantly albumins) and is excreted equally by bile and urine.^[23]^ It is reasonable to assume that a drug with such pharmacokinetics may increase the likelihood of a drug side effect if its clearance is reduced, and in this case, coagulopathy occurs.

Due to the presence of a cardiac device, elevated inflammatory parameters, and a positive blood culture for *S aureus*, a fundamental suspicion of IE was raised. Transthoracic echocardiography showed no signs of valvular vegetations, but according to the guidelines of the European Society of Cardiology, transesophageal echocardiography should be performed if IE is suspected. Initially, the patient did not agree to undergo transesophageal echocardiography, and in the further course of hospital treatment, she was not suitable for the examination due to the development of hemorrhagic shock.^[4]^ The use of antibiotic (antistaphylococcal) therapy for a total of 6 weeks and with the removal of a metal foreign body (pacemaker battery), reduced the possibility of IE to a minimum. Since no rhythm disturbances or asystolic pauses were recorded on telemetry, and with sensing of 95% and pacing of 5%, the patient no longer had indications for the reintroduction of the permanent pacemaker. Furthermore, antibiotic treatment was continued with imipenem due to suspected secondary bacterial infection (arising postoperatively), with the addition of vancomycin due to diarrhea (without the presence of *Clostridium difficile* antigen in the stool). Control blood cultures were sterile before discharge from the hospital.

No significant interactions between rifampicin and previous chronic therapy were observed using the drug-interaction checker available on the web.^[24]^ Two potentially significant drug interactions with rifampicin are a reduction in the effect of atorvastatin, which was overcome by increasing the dose to 40 mg, and that with repaglinide, which was avoided by administering short-acting insulin according to glycemic values, before main meals.^[25]^

The exact mechanism, in this case, was crucial and dominant in the development of coagulopathy is impossible to determine. We assume that this is a multifactorial effect caused by the activation of the immune system with the presence of comorbidities such as terminal uremia, cardiac (metal) device, and infection. It is expected that rifampicin-induced coagulopathy is more common than reported cases in the literature, but due to the difficult recognition and complex pathophysiological mechanism of the development of coagulation disorders, it remains unrecognized.

## 4 Conclusion

Given the unpredictability and infrequent occurrence of rifampicin-induced coagulopathy in clinical practice, clinicians should be aware of the possibility of bleeding or drug-induced thromboembolic events. Moreover, taking into account comorbidities such as liver disease, hypoalbuminemia, malignancies, chronic or acute infectious diseases, and previously recorded allergic reactions to the drug, can greatly help in the early detection of coagulation disorders, timely treatment, and achieving a positive final outcome for the patient.

## Author contributions

**Conceptualization:** Domagoj Vučić, Katica Cvitkušić-Lukenda, Blaženka Miškić.

**Data curation:** Ivica Dunđer.

**Investigation:** Domagoj Vučić, Katica Cvitkušić-Lukenda, Ivica Dunđer, Krešimir Gabaldo, Marijana Knežević-Praveček.

**Methodology:** Krešimir Gabaldo, Marijana Knežević-Praveček.

**Resources:** Domagoj Vučić.

**Supervision:** Blaženka Miškić.

**Writing – original draft:** Domagoj Vučić.

**Writing – review & editing:** Katica Cvitkušić-Lukenda, Ivica Dunđer, Krešimir Gabaldo, Marijana Knežević-Praveček, Blaženka Miškić.
